# 360° CZT-SPECT/CT cameras: ^99m^Tc- and ^177^Lu-phantom-based evaluation under clinical conditions

**DOI:** 10.1186/s40658-024-00684-6

**Published:** 2024-10-24

**Authors:** Christopher Hoog, Pierre-Malick Koulibaly, Nicolas Sas, Laetitia Imbert, Gilles Le Rouzic, Romain Popoff, Jean-Noël Badel, Ludovic Ferrer

**Affiliations:** 1 Medical Physics Department, Institut Godinot Comprehensive Cancer Center, Reims, France; 2 Department of Diagnostic Radiology and Nuclear Medicine, Antoine Lacassagne Comprehensive Cancer Center, Université Nice-Côte d’Azur, 33 Avenue de Valombrose, 06189 Nice, France; 3 Department of Medical Physics, Jean Perrin Comprehensive Cancer Center, 63000 Clermont-Ferrand, France; 4 Department of Nuclear Medicine and Nancyclotep Imaging Platform, CHRU-Nancy, Université de Lorraine, 54000 Nancy, France; 5 Nuclear Medicine Department, CHU Orleans, 14 Avenue de l’Hôpital, 45100 Orleans, France; 6 Department of Medical Physics, Georges-François Leclerc Cancer Center, 1 Rue du Professeur Marion, 21000 Dijon, France; 7 ICMUB, UMR 6302, CNRS, Dijon, France; 8 Centre de Lutte Contre le Cancer Léon-Bérard, CREATIS CNRS UMR 5220 INSERM U 1044, Université de Lyon, INSA-Lyon, Lyon, France; 9 Medical Physics Department, ICO René Gauducheau, Saint Herblain, 44805 France; 10 CRCINA, UMR 1232, INSERM, Nantes, France

**Keywords:** Nuclear medicine, Molecular imaging, 360° CZT-SPECT/CT cameras, Phantom study, Quantification

## Abstract

**Purpose:**

For the first time, three currently available 360° CZT-SPECT/CT cameras were compared under clinical conditions using phantom-based measurements.

**Methods:**

A ^99m^Tc- and a ^177^Lu-customized NEMA IEC body phantom were imaged with three different cameras, StarGuide (GE Healthcare), VERITON-CT versions 200 (V200) and 400 (V400) (Spectrum Dynamics Medical) under the same clinical conditions. Energy resolution and volumetric sensitivity were evaluated from energy spectra. Vendors provided the best reconstruction parameters dedicated to visualization and/or quantification, based on their respective software developments. For both ^99m^Tc- and ^177^Lu-phantoms, noise level, quantification accuracy, and recovery coefficient (RC) were performed with 3DSlicer. Image quality metrics from an approach called “task-based” were computed with iQMetrix-CT on ^99m^Tc visual reconstructions to assess, through spatial frequencies, noise texture in the background (NPS) and contrast restitution of a hot insert (TTF). Spatial resolution indices were calculated from frequencies corresponding to TTF_10%_ and TTF_50%_.

**Results:**

Despite the higher sensitivity of VERITON cameras and the enhanced energy resolution of the V400 (3.2% at 140 keV, 5.2% at 113 keV, and 3.6% at 208 keV), StarGuide presents comparable image quality. This highlights the need to differentiate sensitivity from count quality, which is influenced by hardware design (collimator, detector block) and conditions image quality as well as the reconstruction process (algorithms, scatter correction, noise regulation). For ^99m^Tc imaging, the quantitative image optimization approach based on RC_mean_ for StarGuide versus RC_max_ for V200 and V400 systems (RC_mean_/RC_max_: 0.9/1.8; 0.5/0.9; 0.5/0.9 respectively—Ø37 mm). SR_TB10/50_ showed nearly equivalent spatial resolution performances across the different reconstructed images. For ^177^Lu imaging, the 113 keV imaging of the V200 and V400 systems demonstrated strong performances in both image quality and quantification, while StarGuide and V400 systems offer even better potential due to their ability to exploit signals from both the 113 and 208 keV peaks. ^177^Lu quantification was optimized according to RC_max_ for all cameras and reconstructions (1.07 ± 0.09—Ø37 mm).

**Conclusions:**

The three cameras have equivalent potential for ^99m^Tc imaging, while StarGuide and V400 have demonstrated higher potential for ^177^Lu. Dedicated visual or quantitative reconstructions offer better specific performances compared to the unified visual/quantitative reconstruction. The task-based approach appears to be promising for in-depth comparison of images in the context of system characterization/comparison and protocol optimization.

## Background

The introduction of cadmium-zinc-telluride (CZT) semi-conductor detectors during the 2000s led to the first major single photon emission computed tomography (SPECT) technological breakthrough since the initial scintillation-based model developed by Hal Anger in 1957 (A-SPECT) [1].

Due to CZT manufacturing costs and detection limited to low energy photons, commercialization of CZT cameras began with small field of view cameras dedicated to cardiology applications (c-CZT). In 2007, D-SPECT (Spectrum Dynamics Medical (SDM)) and 2009, Discovery NM530c (General Electric Healthcare (GEHC)), were introduced. Whilst the NM530c camera is equipped with fixed multi-pin hole collimators, D-SPECT is designed with several block detectors having parallel-hole collimator and swiveling detector motion to focus on the heart area. The benefits of these technologies in terms of sensitivity, spatial and energy resolution, resulted in improved image quality and the ability to perform dual isotope imaging [2]. In 2016, GEHC introduced the Discovery NM/CT 670, a general-purpose dual head digital SPECT system equipped with CZT flat panels (CZT-SPECT). With the ability to image isotopes with energies up to 250 keV, this camera enabled access to a wider range of conventional nuclear medicine (NM) clinical examinations. CZT technology offered significant improvements in the dose-image quality trade-off, particularly in the context of planar imaging for which a higher diagnostic performance was demonstrated at lower injected activity or acquisition duration [3–5]. However, some limitations of conventional cameras remained, such as collimator management, photon loss due to geometry and step-by-step whole-body 3D imaging. In 2017 and 2021 respectively, VERITON (SDM) and StarGuide (GEHC) general-purpose systems were introduced to the market as a new design of CZT camera called 3D-ring or 360° CZT-SPECT (360CZT-SPECT) aiming to fully exploit CZT performances and overcome dual head design limitations.

These new systems consist of twelve detector arms evenly distributed over 360° with a triple detector motion mechanism combining a radial linear motion towards the patient, a ring rotation with up to six angular positions to improve angular sampling and a swiveling motion for better focusing. At the tip of each arm, CZT crystal detectors and their fixed collimator will swivel whilst scanning [6, 7]. This scanning is repeated for each bed position. The fixed collimators were designed to be efficient over a wide energy range depending on the models.

Since the introduction of cardiology-dedicated, flat panel and ring shaped CZT camera designs, multiple phantom studies have been published to assess and compare their performances, sometimes in comparison to conventional systems [7–12]. Additionally, clinical studies have validated performances of such cameras for clinical usage [13–18]. For example, Desmonts et al. showed that 360CZT-SPECT had superior sensitivity, better energy resolution and image contrast than A-SPECT camera, whereas spatial resolution remained similar. The study concluded that the “introduction of this new technology may change current practices in nuclear medicine such as decreasing acquisition time and activity injected to patient” (phantom and clinical study, ^99m^Tc, ^123^I, ^201^Tl, ^111^In) [7]. Carsuzaa et al. [13] showed no significant difference in left ventricular volumes and left and right ventricular ejection fractions between c-CZT- and 360CZT-SPECT performing gated tomographic radionuclide angiography (clinical study, 50 patients). Even the scan intolerance rate due to claustrophobia was explored between A-SPECT and 360CZT-SPECT and showed no significant difference in patient experience [19]. These studies suggested 360CZT-SPECT could replace and even surpass both A-SPECT and c-CZT.

Image quality aside, absolute quantification is one of the key remaining challenges of conventional nuclear medicine. Through studies involving several multi-vendor A-SPECT cameras, Peters et al. highlighted a need for standardization as “close agreement between vendors and sites is key for multi-center dosimetry and quantitative biomarker studies” [20, 21]. Three recent phantom studies aimed to assess the quantitative performances of 360CZT-SPECT cameras within the context of 177-Lutetium (^177^Lu) PSMA dosimetry calculation. Danieli and al. [22] and Vergnaud et al. [23] validated the quantification performances of StarGuide and VERITON systems respectively when using optimized reconstruction protocols. While comparing 360CZT-SPECT to conventional cameras, Nuttens et al. [24] showed slightly lower absorbed dose uncertainties in favor of conventional A-SPECT, but all studies agreed this new technology greatly reduced procedure time for whole-body SPECT imaging with around threefold faster scans.

To our knowledge, the three currently available 360° CZT-SPECT/CT cameras have never been compared head-to-head, neither through phantom nor clinical studies. Although NEMA standards are not fully adapted to these cameras (for example, intrinsic mode is unavailable, image sampling is unsuitable for assessing extrinsic spatial resolution), vendor datasheets already exist and have been published [10, 11]. The focus of our study was not on optimization or pure performance assessments. Instead, we aimed to adopt a more clinically oriented approach by performing acquisitions and reconstructions of a customized NEMA IEC body phantom under realistic activity and protocols. The vendors developed their devices and reconstruction parameters using clinical data, particularly in collaboration with the sites selected for this study, Orléans for the StarGuide and Nancy for the VERITON cameras, thereby enhancing the relevance of our research to clinical conditions. The three cameras were evaluated according to different conventional metrics computed from the acquired spectra and the reconstructed images. Also, we took the opportunity to introduce noise power spectrum (NPS) and task transfer function (TTF) allowing deeper assessments of noise texture and clinical spatial resolution respectively. These metrics are notably involved in an approach called “task-based” dedicated to assessing the detectability of a task function through an index called “d-prime”. The task function is defined by choosing the type of signal to detect, called “clinical task”, and entering its diameter and contrast [25, 26]. This approach is currently used to compare or optimize CT imaging protocols should be helpful in nuclear medicine while addressing shortcomings of NEMA standards.

This study focuses on both 99m-Technetium (^99m^Tc), probably the most used isotope in conventional nuclear medicine [27], and ^177^Lu which is an extremely challenging radionuclide in terms of theranostics, quantification and dosimetry [28–34].

## Methods

### Cameras

Three 360CZT-SPECT cameras combined with a computed tomography (CT) scanner were evaluated: StarGuide (GEHC, Haifa, Israel) installed in 2020 at the nuclear medicine department of CHU Orléans (Orléans, France); VERITON 200 (V200) and VERITON 400 (V400) (SDM, Caesarea, Israel) respectively installed in 2018 and 2021 at the department of nuclear medicine of CHRU Nancy (Nancy, France). The overall imaging bore of systems is 70 cm for StarGuide and 80 cm for V200 and V400. The VERITON cameras have a 32 cm axial field of view versus 28 cm for StarGuide. The StarGuide and V400 systems have thicker CZT crystal detectors as opposed to V200 (7.25, 7.3 mm and 6 mm respectively). Whilst StarGuide and V400 can acquire photons above 200 keV, V200 detector electronics limit acquisition to 200 keV. V200 and V400 are equipped with the exact same collimator.

### Phantom

**Fig. 1:**
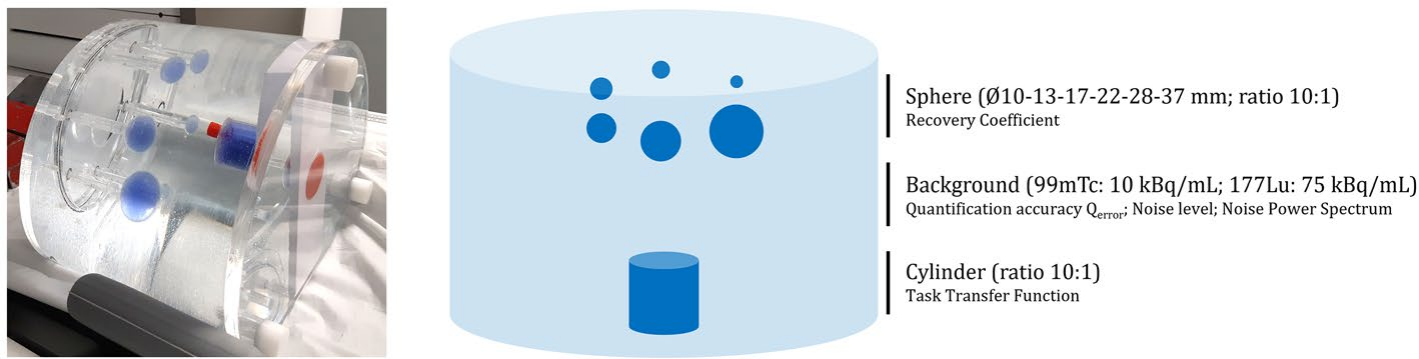
Customized NEMA IEC body phantom.

A customized NEMA IEC body phantom (PTW, Breisgau, Germany) was used for conventional voxel value-based analyses and innovative task-based image quality assessment. The lung insert was removed and replaced with a fillable cylindrical insert (32 mm diameter x 50 mm height), leading to three distinct zones: “sphere,” “background,” and “cylinder” (Fig. 1). The “sphere” section is dedicated to recovery curves assessments. The “background” section is dedicated to quantification accuracy (Q_error_), noise level and noise texture assessments. The cylinder in the third section simulates a hot lesion on which edge spread function measurements helped in quantifying a clinical spatial resolution. For each camera, two phantoms filled with ^99m^Tc and ^177^Lu respectively, were scanned. To mimic clinical conditions, background concentrations of 10 kBq/mL for ^99m^Tc and 75 kBq/mL for ^177^Lu were defined. The spheres and the cylinder were filled with a more concentrated radioactive solution to achieve a 10:1 sphere to background ratio (Table 1) following the stock solution method filling one tenth of the background of the phantom (volume defined by weighted with a precision balance).

**Table 1:** Phantoms preparation.

		StarGuide	V200	V400
^99m^Tc	[A_Background_] (kBq/mL)	10.4	9.6	8.4
	Sphere to background ratio	10.0	10.0	10.0
^177^Lu	[A_Background_] (kBq/mL)	78.8	74.5	74.9
	Sphere to background ratio	9.8	10.0	10.0

### Image acquisition & reconstruction

Positioning of the phantom and scan acquisitions were consistent across all cameras with each phantom positioned flat on the examination table and precisely aligned within the CZT detectors’ central axial field of view. With the StarGuide system, table height was automatically set-up, whilst for VERITON, table height was manually adjusted using laser alignment. Prior to SPECT data acquisition, X-ray scout views and CT scans were performed to validate correct positioning of the phantoms. These scans provided both anatomical imaging and µ-map for attenuation correction (CTAC). Subsequently, ten-minute list mode acquisitions were performed, adhering to routine clinical protocols for both CT imaging and nuclear medicine (NM) data collection, as outlined in Table 2. Energy spectra and ten-minute NM reconstructions were generated from list-mode data files.

**Table 2:** Clinical protocols used for CT imaging and NM acquisitions.

			StarGuide	V200	V400
CT			Helical 0.80 s; Pitch 1.375; 120 kV; 80.0 mA	Helical 0.67 s; Pitch 1; 120 kV; 80.4 mAs	
			CTDI vol.: 4.12 mGy	CTDI vol. : 5.58 mGy	
NM	Duration		10'00'' + 30 s (rotation)	10'00'' + 30 s (rotation)	
	Rotation		4 angles	4 angles	
	^99m^Tc	140 keV	Lower scatter: [114–126] keV Photopeak: [126–154] keV (140±10%)	Lower scatter: [109–130] keV Photopeak: [130–151] keV (140±7,5%)	
	^177^Lu	113 keV	Lower scatter: [92.1–100.9] keV Photopeak: [101.7–124.3] keV (113 ± 10%) Upper scatter: [125.1–134.2] keV	Lower scatter: [79–101.6] keV Photopeak: [101.6–124.2] keV (112.9 ± 10%) Upper scatter: [124.2–146.8] keV	
		208 keV	Lower scatter: [175.8–194.3] keV Photopeak: [195.5–220.5] keV (208±6%)	–	Lower scatter: [161.6–192.8] keV Photopeak: [192.8–224] keV (208 ± 7.5%) Upper scatter: [224–255] keV

In the clinical setting, manufacturers provide the ability to perform reconstructions that are optimized for diagnostic quality and/or enhanced quantitative accuracy. We therefore requested both types of reconstructions, depending on the specifications provided by respective manufacturers. In the case of the ^177^Lu-phantom, it's worth mentioning that both 113 keV and 208 keV peaks were accessible for StarGuide and the V400 systems, whereas the V200 camera was constrained to the 113 keV peak only.

All 2.46 mm voxel size reconstructions were performed using ordered subset expectation maximization (OSEM) or QClear, a Bayesian penalized likelihood (BPL) reconstruction algorithm using block-sequential regularized expectation maximization (BSREM) [35]. CTAC was applied on every reconstruction. Optional scatter correction (nSC/SC) was applied using dual/triple energy window methods (DEW/TEW) depending on isotope and peak: DEW for ^99m^Tc 140 keV (GEHC) and ^177^Lu 208 keV (GEHC); TEW for ^177^Lu 113 keV (GEHC-SDM) and 208 keV (SDM-V400). Point Spread Function Recovery (PSFR), here called Resolution Recovery (RR) was systematically used for GEHC reconstructions and optionally used for SDM reconstructions. It is worth noting that SDM’s RR is based on the system response to a point source scanned successively in air and in a scatter media. This empirical model will therefore correct aberrations due to the system's elements (collimator, crystal…) but as the model is based on actual experimental acquisition, will also include Compton scattering effects. In a first approximation, this scattering correction is robust enough for ^99m^Tc for which most user opt to apply RR only. For ^177^Lu, for which the scatter fraction is more important due to down scatter from the 208 keV peak, scatter correction included in RR results in a more pronounced under correction and most user comply with SDM guidelines to use the triple energy window-based scatter correction. Therefore, to differentiate the methodology for SC is always specified either as SC(DEW/TEW) or SC(RR). To avoid over-correction, both types cannot be applied together. Noise regulation (NR) is performed through relative difference penalty (RDP) [35] and Clarity3D for StarGuide and intra- and post-iteration filtering for VERITON cameras.

For ^99m^Tc, distinct reconstructions dedicated to visualization and quantification were provided for all three cameras. The visual reconstruction for StarGuide was based on QClear AC nSC NR, while the quantitative reconstruction was based on OSEM AC SC(DEW) nNR, resulting in a significant difference in image quality. Both visual and quantitative reconstructions for the VERITON systems were OSEM AC SC(RR) NR. The V200 and V400 quantitative reconstructions differed from the visual ones only by the number of subsets (8 vs. 32).

For ^177^Lu, StarGuide provided visual/quantitative reconstructions for the 113 keV and 208 keV peaks, based on QClear AC SC(TEW/DEW) NR. To fully exploit the recorded data, GE recommend generating an image as the voxel-wise arithmetic sum of the images obtained from the two peaks. For the 113 keV peak of V200 and V400, the visual image was AC nSC NR, while the quantitative image was AC SC(TEW) NR. V400 did not provide a separate image for the 208 keV peak but offered a combined 113-208 keV image considered both visual and quantitative. This combined image is the average of a reconstruction of the 113 keV peak AC SC(TEW) NR and a reconstruction of the 208 keV peak AC SC(RR) NR (independent images were not available for this study).

### Quantification

All injected activities were measured using on-site dose calibrators: Capintec CRC15R in Orléans and Capintec CRC-25 in Nancy. For ^99m^Tc, both dose calibrators were initially certified by the French National Laboratory “Henri Becquerel” (LNHB). For ^177^Lu, vial and syringe calibration factors were established following LNHB guidelines, using a ^177^Lu source provided by Advanced Accelerator Applications (Saint Genis Pouilly, France). Both dose calibrators are subject to a strict quality insurance policy according to French legislation. Acceptance tests include accuracy, reproducibility, linearity and geometry response. Daily controls consist of background check and constancy of the response with a long half-life check source. The test for constancy of the response is performed monthly with two long half-life check sources. No volume correction factor was calculated for ^177^Lu.

Both manufacturers achieved absolute quantification through a cross-calibration methodology, which involves acquiring data from a cylindrical phantom containing a homogeneous radioactive solution with a known activity concentration, as determined by a dose calibrator. The determination of a calibration factor for each specific isotope is computed as the average count observed in the phantom's uniform background (spherical VOI 80% phantom diameter) divided by the time duration and the activity concentration at the time of acquisition. The calibration factors are defined for quantitative imaging only and involve the dedicated reconstruction algorithm.

For the ^177^Lu isotope, StarGuide produces images for both the 113 keV and 208 keV energy peaks, requiring the computation of distinct calibration factors for each of these peaks. In contrast, with the V200 and V400 systems, a calibration factor is solely computed for the 113 keV energy peak. The V400 system enables quantitative imaging by combining data from both the 113 keV and 208 keV peaks. To reach consistency in the average signal within the homogeneous background, a normalization factor for the 208 keV peak is derived from the 113 keV image. All calibration factors were verified, and updated if required, prior to performing the phantom acquisitions for this study.

### Analysis

#### Energy resolution and volumetric sensitivity from spectra

For a relevant comparison, the energy spectra obtained from raw data were resampled with a 1 keV step and normalized in kcps/(MBq/mL) considering the 600 s acquisition duration and the activity concentration at the time of acquisition. The energy resolution of each exploitable peak was calculated as the ratio of the full width at half-maximum (FWHM) to the measured energy peak. Furthermore, a volumetric sensitivity value was calculated by integrating the counts over the photopeak energy window normalized by acquisition duration and activity concentration.

#### Image quality and quantification accuracy assessment from reconstructed images

**Fig. 2:**
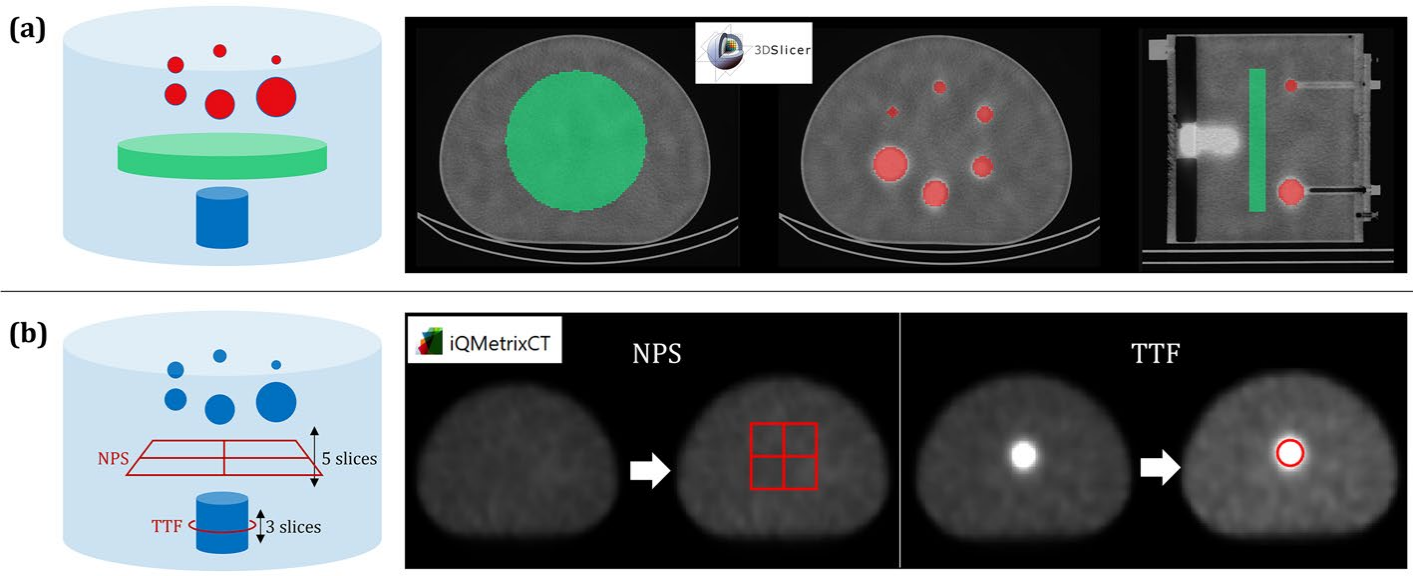
Illustration of voxel value-based metrics measurements from 3DSlicer (**a**), task-based quality image assessment with iQMetrix-CT (**b**).

Quantitative analyses using conventional metrics were conducted using the 3DSlicer (v5.2.2) software. After processing a NM-CT registration (X-Y-Z translations), spherical volumes of interest (VOI) and a cylindrical VOI (about 250 cm^3^) were defined on the CT images to match the internal diameter of the spheres (Fig. 2a red VOIs) and the homogeneous background respectively (Fig. 2a green VOIs). From NM reconstructions, the maximum and mean values of each sphere were calculated, as well as the mean and standard deviation values in the homogeneous background. Using those measurements, the following metrics were calculated (Table 3):

- Noise level based on the standard deviation and mean voxel value measured in the background.
- Q_error_ based on the mean voxel value measured in the background and the true activity concentration in the background.
- Recovery coefficient of each sphere based on the mean (RC_mean_) or maximum (RC_max_) voxel value measured for each sphere, the mean voxel value measured in the background, and the true activity concentrations both in the spheres and background.

**Table 3:** Formula/metrics and analysis software.

	Formula/Metric	Phantom section	Software	Images
Noise	$\text{Noise level }\left(\text{\%}\right)=100*\frac{{\text{SD}}_{\text{background}}}{{\text{Mean voxel value}}_{\text{background}}}$	Background	3DSlicer	All images
	NPS; f_mean_; f_max_	Background	iQMetrix-CT	All images
Spatial Resolution	TTF; SR_TB10_ =1/f(TTF_10%_); SR_TB50_ =1/f(TTF5_0%_)	Cylinder	iQMetrix-CT	^99m^Tc—Visual
Quantification	${\text{Q}}_{\text{error}} (\text{\%})=100*\left(\frac{{\text{Mean signal }}_{\text{background}}}{{\text{Activity concentration}}_{\text{background}}}-1\right)$	Background	3DSlicer	^99m^Tc—Quant ^177^Lu—Quant. and Visual/Quant.
	$\text{Recovery coefficient }=\frac{\frac{{\text{Max or Mean voxel value}}_{\text{sphere}}}{{\text{Mean voxel value}}_{\text{background}}}}{\frac{{\text{Activity concentration}}_{\text{sphere}}}{{\text{Activity concentration}}_{\text{background}}}}$	Sphere & Background	3DSlicer	^99m^Tc—Quant ^177^Lu—Quant. and Visual/Quant.

**Fig. 3:**
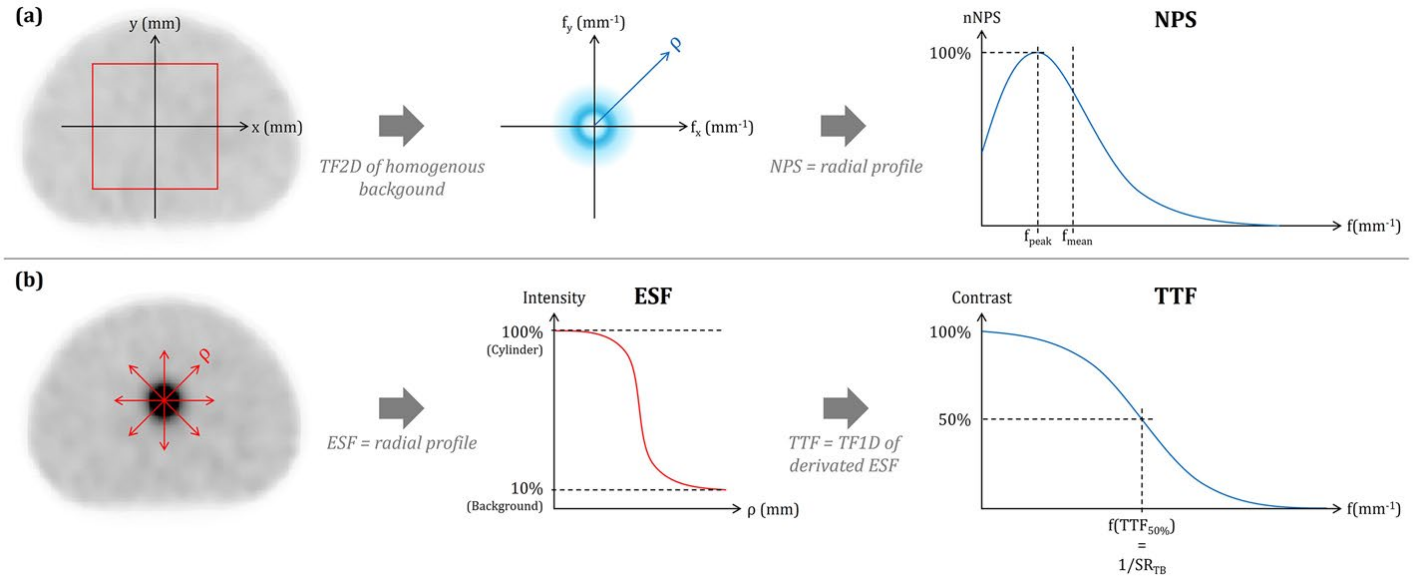
Illustration of NPS (**a**) and TTF (**b**) estimations from phantom homogeneous background and a cylindrical insert mimicking a lesion uptake.

Inspired from the “task-based” image quality assessment approach currently used to compare or optimize CT imaging protocols, we introduced NPS and TTF to assess noise texture in the background and contrast restitution of a cylindrical insert mimicking an anatomical specific density (CT) or in our case a lesion uptake. From the radial profile of the 2D Fourier transform of a homogeneous image, the NPS quantifies both noise amplitude and texture (Fig. 3a) and helps identify structures and patterns in homogeneous images [36]. TTF is computed as the 1D Fourier transform of the derivative edge spread function (ESF) obtained between the center of the cylinder and the background (Fig. 3b). NPS and TTF were performed using the iQMetrix-CT software (v1.1) [25] (Fig. 2b, Table 3):

- The normalized NPS (_n_NPS) curves were calculated from 4 square regions of interest (automatically sized) duplicated on 5 consecutive axial slices within the homogeneous region. This allowed for a comparison of noise texture, particularly in terms of peak frequency (f_peak_) and average frequency (f_mean_).
- The TTF was evaluated from a circular region of interest (automatically placed) duplicated on 3 consecutive axial slices around the cylindrical insert. According to AAPM Report 223 [26], a CNR value greater than 15 between the cylinder and background must be achieved for a meaningful TTF. The TTF calculation was performed based on an unconditioned and unsymmetrized Edge Spread Function (ESF) obtained by the "pixel distance" method, and a filtered Line Spread Function (LSF) using the Hann filter. Spatial resolution indices based on the clinical task (SR_TB_) were calculated as the inverse of the frequencies corresponding to 10% and 50% of the TTF maximum intensity (Table 3).

Noise level was assessed on every reconstruction. Q_error_ was quantified on dedicated quantitative reconstructions (quant and visual/quant.). RC curves were only deducted from quantitative and visual/quantitative images. TTF was only applied on the ^99m^Tc visual reconstructions as it was the only case that satisfied a CNR superior to 15 between insert and background.

## Results

### Energy resolution & volumetric sensitivity from energy spectra

**Fig. 4:**
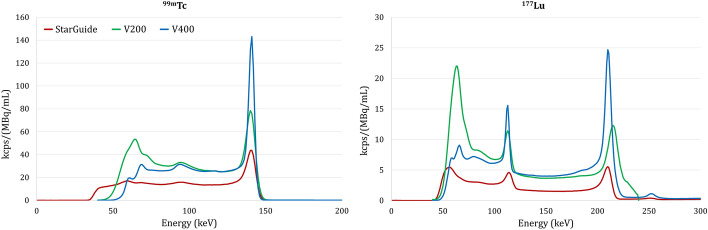
Energy spectra recorded from the acquisitions of ^99m^Tc (left) and ^177^Lu (right) NEMA IEC body phantoms. The energy spectra were resampled with a 1 keV step and normalized in kcps/(MBq/mL).

Figure 4 shows the resampled spectra and Table 4 the results of their analysis. All three cameras exhibited a peak around 60 keV, originating from the tungsten collimator fluorescence. However, this peak was higher for V200, especially for the ^177^Lu phantom. It should be noted that the apparent peak of V200 does not represent the information from 208 keV photons; all the information measured beyond 200 keV is indeed re-indexed between 200 and 250 keV.

**Table 4:** Energy spectra analysis.

	StarGuide			V200		V400		
Theoretical peak (keV)	140.5	113.0	208.0	140.5	113.0	140.5	113.0	208.0
Measured peak (keV)	140.5	114.0	210.0	140.7	113.4	141.3	113.2	210.8
Delta peak (keV)	0.0	1.0	2.0	0.2	0.4	0.8	0.2	2.8
FWHM (keV) *	8.8	11.7	12.1	8.2	10.2	4.5	5.9	7.6
Measured energy resolution (%)	6.2	10.3	5.7	5.8	9.0	3.2	5.2	3.6
NEMA energy resolution (%)—Datasheet [10;11]	5.4	7.5	4.6	5.7	7.5	3.8	4.5	3.5
Measured volumetric sensitivity (kcps/(MBq/mL))	499	72	74	764	170	919	165	285
NEMA volumetric sensitivity (kcps/(MBq/mL)) —Datasheet [10;11]	542	–	–	≥ 610	≥ 135	≥ 760	≥ 115	≥ 185

^*^Because of the scatter level on StarGuide and V200, the FWHM of 113 keV peak (177Lu) was estimated as the double of the superior part of the FWHM

For ^99m^Tc, peak position was measured on the three cameras at 140.5 keV, 140.7 keV and 141.3 keV on StarGuide, V200 and V400 respectively. For ^177^Lu, all three systems measured the 113 keV peak within 1 keV accuracy and the 208 keV at 210 keV on StarGuide and 210.8 keV on V400. These results do not reflect an intrinsic characteristic of the cameras as they depend on environmental factors and peaking calibration.

In terms of energy resolution, StarGuide achieved a 6.2%, 10.3% and 5.7% energy resolution for peak 140 keV (^99m^Tc), 113 keV and 208 keV (^177^Lu) respectively. V200 achieved a 5.8% and 9.0% energy resolution for peak 140 keV (^99m^Tc) and 113 keV (^177^Lu) respectively. V400 achieved a 3.2%, 5.2% and 3.6% energy resolution for peak 140 keV (^99m^Tc), 113 keV and 208 keV (^177^Lu) respectively.

StarGuide offered a volumetric sensitivity of 499, 72 and 74 kcps/(MBq/mL) at 140 keV (^99m^Tc), 113 keV and 208 keV (^177^Lu) respectively. V200 offered a volumetric sensitivity of 764 and 170 kcps/(MBq/mL) at 140 keV (^99m^Tc) and 113 keV (^177^Lu) respectively. V400 offered a volumetric sensitivity of 919, 165 and 285 kcps/(MBq/mL) at 140 keV (^99m^Tc), 113 keV and 208 keV (^177^Lu) respectively.

### Image quality and quantification accuracy assessment from reconstructed images

#### ^99m^Tc

**Fig. 5:**
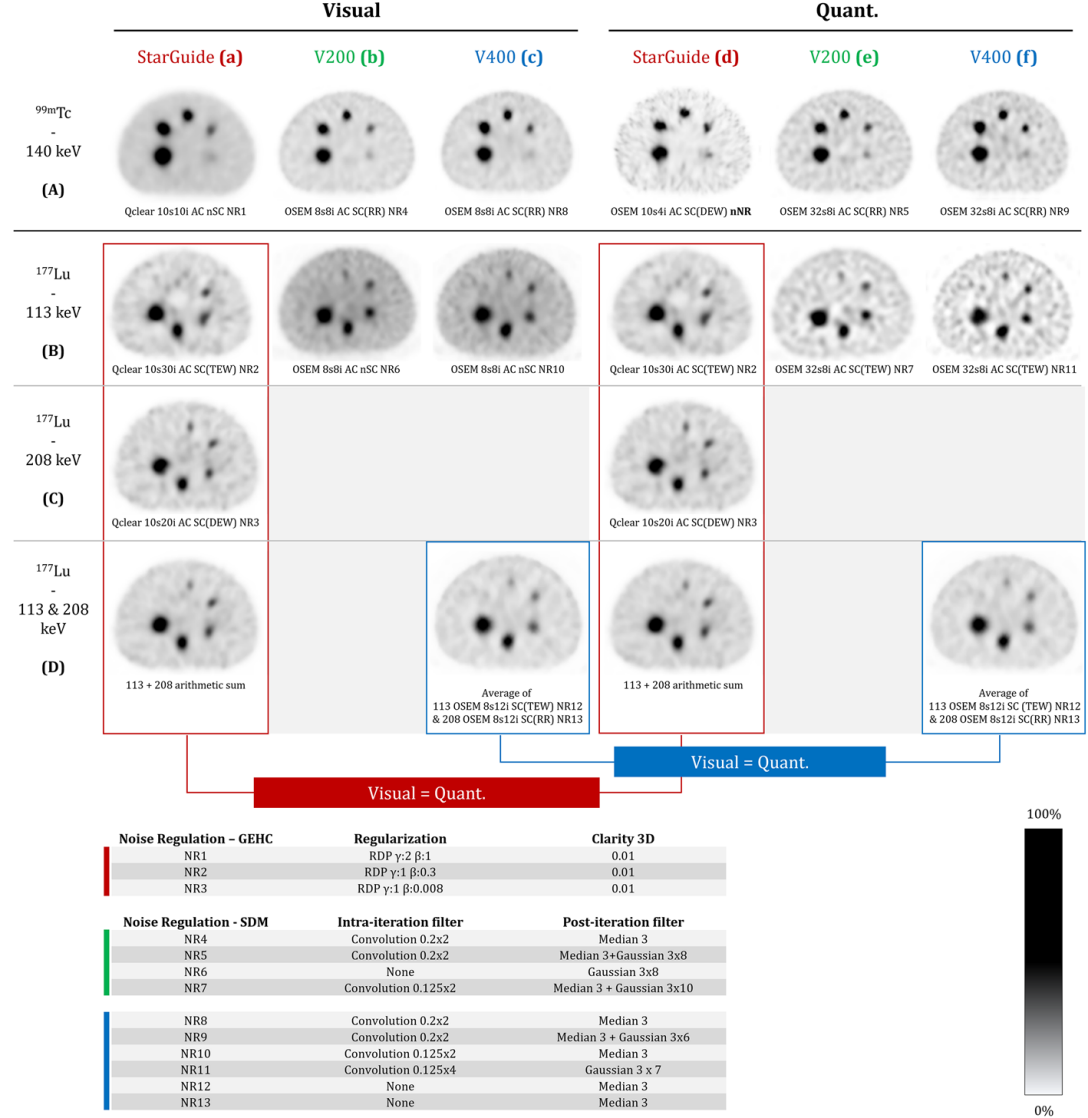
Visual (**a**–**c**) and quantitative (**d**–**f**) reconstructions from ten-minute acquisitions of the ^99m^Tc (**A**) and ^177^Lu (**B**–**D**) NEMA IEC body phantoms (spheres mid-plane) on StarGuide, V200 and V400 cameras. The images are displayed with a contrast set at 50% of the maximum. The algorithm for each image is specified (OSEM: Ordered Subset Expectation Maximization) and reconstruction parameters (AC: Attenuation Correction; SC: Scatter Correction; RR: Resolution Recovery; NR: Noise Regulation; n-: non-; DEW: Double Energy Window; TEW: Triple Energy Window).

With all three cameras, the four largest spheres are clearly resolved on both visual and quantitative reconstructions, the fifth sphere appears with low contrast but with a better definition on V200, and the smallest sphere was not visible (Fig. 5Aa–f). When focusing on visual reconstructions, VERITON cameras offered a more conventional noise texture and relevant spheres size whereas the background appeared smoother and the spheres larger on StarGuide (Fig. 5Aa–c). Regarding quantitative reconstructions, all images appeared noisier than visual ones. With StarGuide we observed a difference in background pattern between the center and the phantom’s edges, as well as deformed spheres (Fig. 5Ad), whilst V200 and V400 exhibited the same background texture and appearance of the spheres (Fig. 5Ae–f) on quantitative and visual images.

**Fig. 6:**
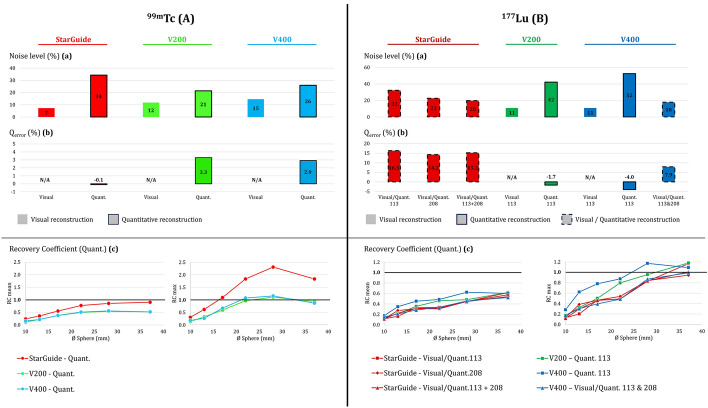
Conventional metrics for ^99m^Tc (**A**) and ^177^Lu (**B**) phantoms: Noise level (**a**) and Q_error_ (**b**) were measured in the homogeneous background section. RC mean and max curves (**c**) were displayed for quantitative reconstructions. On histograms, black outlines illustrate quantitative reconstructions (dotted outlines for visual/quant. reconstructions). N/A stands for Not Applicable.

StarGuide led to the lowest and highest noise level in the background for visual QClear and quantitative OSEM reconstructions, with values of 7% and 34% respectively. V200 allowed a slightly lower noise level compared to V400 for both visual (12% vs. 15%) and quantitative (21% vs. 26%) reconstructions. VERITON noise level difference between visual and quantitative reconstructions was less accentuated than on StarGuide (Fig. 6Aa)

StarGuide offered a quantification accuracy within 0.1% in the background of the OSEM SC quantitative image. Quantification error was about 3.3% and 2.9% for quantitative reconstructions of V200 and V400 cameras respectively (Fig. 6Ab).

As shown with RC curves obtained from quantitative reconstructions (Fig. 6Ac), on StarGuide we measured RC_mean_ values of 0.77, 0.86, and 0.90 for the Ø22 mm, Ø28 mm, and Ø37 mm spheres respectively and RC_max_ exceeded 1.00 for the Ø17 mm sphere, ranging from 1.83 to 2.31 for larger spheres. The V200 and V400 cameras showed similar performances, converging to 0.5 (RC_mean_) and 1.0 (RC_max_) from the Ø22 mm sphere.

**Fig. 7:**
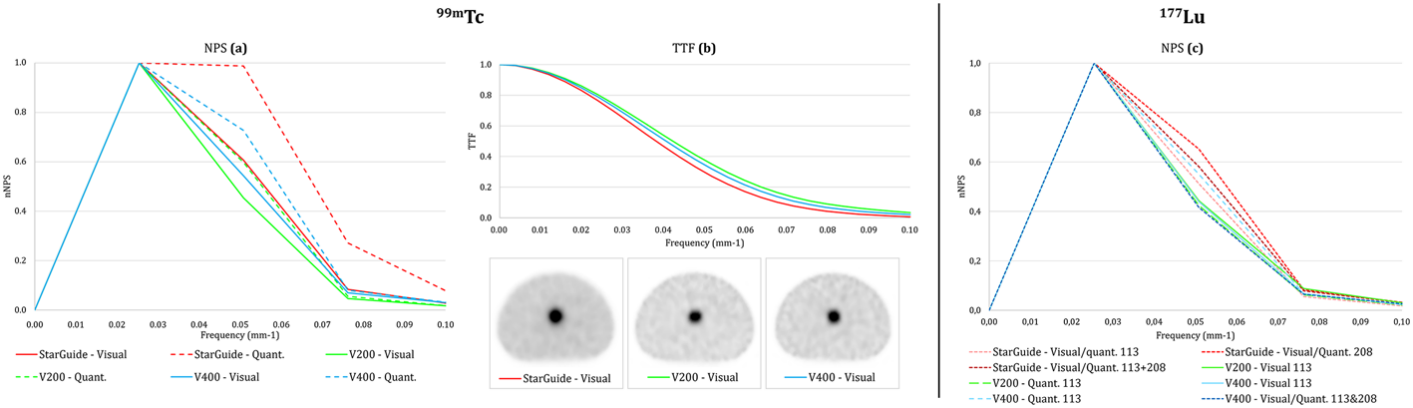
Noise power spectrum (NPS) (**a**) and task transfer function (TTF) (**b**) from the ^99m^Tc-phantom and NPS (**c**) from the ^177^Lu- phantoms. NPS and TTF are computed from the background and cylinder section of the phantom respectively.

The six _n_NPS curves of both visual and quantitative reconstructions from the three cameras had the same peak frequency at 0.025 mm^-1^. The average frequencies between 0.037 and 0.041 mm^-1^ also indicated an equivalent noise texture across all reconstructions, except for the quantitative reconstruction from StarGuide, which showed a different visual aspect translated with an average frequency of 0.047 mm^-1^ (Fig. 7a)

The TTF curves of the three visual reconstructions were quite similar. We computed SR_TB10_/SR_TB50_ values of 14.7/26.2, 12.9/23.7, and 13.7/24.6 mm for the StarGuide, V200, and V400 respectively (Fig. 7b).

#### ^177^Lu

For 113 keV peak imaging, the visual/quantitative reconstructions from StarGuide presented artifacts and only allowed to discern four distorted spheres (Fig. 5Ba,d). The nSC nRR visual images from the V200 and V400 displayed better quality with a more homogeneous background and better geometric restitution of the five visible spheres (Fig. 5Bb, c). Due to the scatter correction, quantitative images of V200 and V400 appeared noisier, but still displayed five visible spheres with a better contrast (Fig. 5Be, f).

The StarGuide camera was able to generate a 208 keV energy window centered image without background artifact where all five spheres were visible (Fig. 5Ca, d).

The StarGuide and V400 generated visual/quantitative images combining both 113 keV and 208 keV peaks (Fig. 5D). These images had a comparable image quality, with a noise—detectability tradeoff allowing the five spheres to be visualized with a homogeneous background.

StarGuide’s visual/quantitative reconstructions led to a noise level of 32% and 23% for 113 keV and 208 keV imaging respectively. V200 and V400 reconstructions for 113 keV peak, led to the same noise level of 11% for visual images and 42% (V200)–52% (V400) for quantitative images. The StarGuide reconstruction obtained from the voxel-wise arithmetic sum of the images of the two peaks led to a noise level of 20%, which is close to the 18% obtained for the visual/quantitative 113–208 keV combined reconstruction from the V400 camera (Fig. 6Ba).

On the three StarGuide reconstructions (113 keV, 208 keV and 113-208 keV), we observed a quantitative overestimation of almost 15% in the background. V200 and V400 113 keV dedicated quantitative reconstructions led to a Q_error_ of − 1.7% and − 4.0% respectively, whilst we measured a Q_error_ of 7.9% on the V400 combined 113–208 keV visual/quantitative reconstruction (Fig. 6Bb).

For visual and visual/quantitative reconstructions (StarGuide 113 keV, 208 keV and 113-208 keV; V400 113 and 208 keV), the recovery curves were quite close to each other, increasing between Ø10 mm and Ø37 mm from 0.12±0.02 to 0.56±0.40 and 0.14±0.02 to 1.03±0.10 for RC_mean_ and RC_max_ values respectively. The 113 keV reconstructions of the V200 and V400 dedicated quantitative images offered better accuracy with regards to the 1.00 SUV target value, especially on all the intermediate spheres (Fig. 6Bc).

The _n_NPS curves showed quite homogeneous noise textures between the reconstructions with the same peak frequency of 0.025 mm^-1^ and mean frequencies between 0.038 and 0.041 mm^-1^. However, we observed a trend with the curves of the VERITON cameras which looked hollower towards the low frequencies versus the more rounded curves of the StarGuide, thus including higher frequencies (Fig. 7c).

## Discussion

Through an original phantom-based study, we established a twofold comparison of StarGuide, V200 and V400 360CZT-SPECT cameras. The first comparison criterion, based on energy spectra analysis, showed VERITON cameras were more sensitive than StarGuide and V400 provided enhanced energy resolution. The second one, based on reconstructed images using clinical protocols, demonstrated the potential of the cameras in terms of image reconstruction and highlighted the different approaches to quantification of ^99m^Tc.

### Customized phantom design

The introduction of a customized NEMA IEC body phantom where the cold lung was replaced by a hot cylinder enabled both conventional and innovative image quality and quantification accuracy assessments (Fig. 1). We improved this approach from a previous study comparing OSEM and xSPECT algorithms (Siemens Healthineers, Erlangen, Germany) [37], where TTF was only performed from the mid-plane of the Ø37 mm sphere. The introduction of the cylinder centered in the field of view enabled computation of the TTF from several slices without axial partial volume effect and limiting target deformation. According to SDM ^99m^Tc datasheets [11], spatial resolution in presence of scatter medium is less than 4.5 mm so we assume our 32 mm diameter cylinder was not significantly impacted by transaxial partial volume effect. However, measurement reproducibility and influence of the cylinder diameter in the relevance of TTF should be studied further. Bailey et al. [38] recently introduced a 70 mm diameter cylinder, compatible with NEMA IEC body phantom, dedicated to assessing the quantification accuracy of long period nuclides. This cylinder could be more adapted to TTF measurements, especially considering ^177^Lu and medium energy collimator that degrades spatial resolution. Moreover, the activity concentration within the cylinder should be increased to ensure a CNR higher than 15, which was not reached for these ^177^Lu-phantom acquisitions.

### Energy resolution and system volumetric sensitivity from spectra

Analyses of the energy spectra showed the energy resolution of StarGuide and V200 were equivalent, but twice higher for V400 (Fig. 4, Table 4). While StarGuide and V400 have similar thick CZT crystals (within 0.05 mm), the higher energy resolution of V400 could be explained by different parameters such as crystal growing technique, whole detector bloc design, high voltage value or electronic signal processing.

The lower volumetric sensitivity of StarGuide could not be justified by the 4 cm shorter axial FOV since the phantom entirely fitted within one bed position for the three cameras. Hence, it ostensibly comes from the collimator design between the two vendors associated with the CZT crystal, electronic design, and signal processing. The sensitivity – spatial resolution tradeoff due to collimator design is a foundational concept of SPECT imaging. We were unfortunately unable to assess or compare collimator designs due to non-disclosure of the VERITON collimator specifications by SDM.

Despite different phantoms and measurement conditions, the measured energy resolution and volumetric sensitivity were in accordance with the manufacturers’ datasheets (Table 4) [10, 11] based on NEMA standards, for both ^99m^Tc and ^177^Lu radionuclides. Our results were also relevant with regards to the Desmonts et al. study on V200 system, presenting a 5.5% energy resolution in air and volumetric sensitivity measurement of 65.8 cps/MBq using a uniformly filled with radioactive solution body phantom [7].

SDM has recently released a V300 camera allowing photon detection up to 300 keV and equipped with 5 mm thickness CZT crystals (6 mm and 7.3 mm for V200 and V400 respectively) and the exact same collimator as V200 and V400 cameras, thus demonstrating that crystal thickness is not the only limiting factor in terms of high energy constraints, and that the whole detection chain, including electronic design and signal processing also play a crucial role in terms of detector performances.

### Reconstruction protocol approach

Description of the different reconstructions (Fig. 5) has highlighted the manufacturers’ strategies for providing the best reconstructions dedicated to visualization and/or quantification, based on their respective software developments:

- For ^99m^Tc, GEHC developed separate reconstructions, optimized for visualization (Q.Clear AC nSC) and quantification (OSEM AC SC). Both reconstruction defaults are available in the system for the reader to use per the required clinical task. Alternatively, VERITON cameras offer both visual and quantitative OSEM SC(RR) reconstructions, the second reconstruction having more updates than the first (Fig. 5A).
- For ^177^Lu theranostic images, GE followed the PET approach, generating diagnostic QClear SC images that also allow quantification. On the other hand, SDM proposes an OSEM nSC reconstruction for the 113 keV peak that offers better visualization comfort (lower noise level); and noisier quantitative OSEM SC(RR) reconstruction that offers recovery coefficients closer to the target value of 1.00 (RC_max_). Finally, the combined 113-208 keV visual/quantitative images from StarGuide and V400 provided the best tradeoff between image quality and quantification. If 113 keV images from V200 and V400 could be fully exploited, this study showed it was not the case with StarGuide, for which the 113-208 keV combined image is the best to be exploited (Fig. 5D]).

### Image quality and quantification accuracy assessment from reconstructed images

Image quality is inherently linked with energy resolution and sensitivity, but this relationship is greatly influenced by hardware design (collimator, detector block), which defines the "count quality". Additionally, acquisition and reconstruction parameters also impact image quality. This explains why, when comparing different systems, it is essential to decouple energy and sensitivity results from image quality. For example, the StarGuide, despite having the lowest sensitivity, exhibited the best SNR for visual reconstruction of ^99m^Tc. The aim of this study was to work under clinical conditions that integrate both thin notion of “count quality” and the practical use of the devices.

Analysis of the conventional metrics (noise level, Q_error_, RC) (Fig. 6) highlighted that scatter correction should be disabled for visualization purposes (lower noise level). All quantitative reconstructions involving scatter correction led to an accurate quantification in the background (Q_error_ < 5%) except for a 15% Q_error_ for ^177^Lu-StarGuide reconstructions. However, this last result was not observed in the Danieli et al. [22] study showing that the quantification accuracy of the StarGuide system, although affected by septal penetration, was < 10% for all ^177^Lu reconstructions.

We also demonstrated the importance of analyzing RC curves to understand the impact of the partial volume effect on quantification accuracy based on lesion size. For StarGuide, the ^99m^Tc quantitative reconstruction was optimized according to RC_mean_, as GEHC considers RC_max_ values are too dependent on noise. For other cases, (StarGuide ^177^Lu, VERITON ^99m^Tc and ^177^Lu), we clearly showed the optimization was based on RC_max_ values. In their study comparing ^99m^Tc quantification performances of several A-SPECT cameras, Peters et al. showed median RC value of sphere Ø37 mm converged to 0.9 (RC_mean_) and 1.2 (RC_max_) [20]. Using RC curves, Tran-gia et al. suggested a partial volume effect correction method in the context of quantitative ^177^Lu SPECT/CT imaging [39]. Finally, RC_mean_ values were estimated from CT-based VOI, therefore without considering deformation on metabolic images. Different studies have explored the impact of the segmentation method for the quantification of spheres, organs, or lesions [20, 23, 37].

Concerning innovative metrics (Fig. 7), _n_NPS curves showed a quite equivalent noise texture of the images according to the cameras and reconstructions, except for StarGuide ^99m^Tc quantitative reconstruction. Indeed, the visibly different pattern between the center and the edges of the phantom was restituted through a _n_NPS curve including higher spatial frequencies. TTF and SR_TB_ enabled direct comparison of spatial resolution between the three cameras through a clinical approach. No significant difference was found between the three cameras with SR_TB10_ and SR_TB50_ values ranging from 12.9 to 14.7 and 24.6 to 26.2 mm respectively. The datasheets following NEMA standards point out the three cameras state a “central reconstructed spatial resolution with a scattering medium of less than 4.5 mm” [10, 11]. If datasheets focus on achieving best spatial resolution using a filtered back projection reconstruction algorithm [40], TTF method is a promising approach to computing a more clinical-like spatial resolution. Whilst the potential of innovative metrics has been illustrated here, our final objective would be to achieve the entire task-based image quality assessment approach computing a relevant detectability index d-prime that would surpass the conventional CNR .

### Perspectives

Through the multiple exchanges we had with the manufacturers, we clearly felt 360CZT-SPECT is still a very recent technology with a lot of improvements to come from hardware and software developments. In particular, the actual energy window inherited from conventional A-SPECT should be adjusted to the energy resolution; as an example, the 15% energy window width does not fit the 3.6% improved energy resolution of the V400-208 keV peak (Tables 2 and 4). Also, the PET approach offering a single visual and quantitative image should be preferred. The manufacturers of ring cameras also state that their fixed collimators will not prevent scanning high energy isotopes such as ^131^I, as future software developments will algorithmically correct for septal penetration.

^177^Lu-labelled pharmaceutical guidelines have not yet been updated regarding quality control [40] and dosimetry for this technology. Indeed, EANM committee recommendations for dose estimation are to use 208 keV peak based images from an A-SPECT camera equipped with a medium energy collimator [41]. This appears to be irrelevant when using 360CZT-SPECT cameras as they include a multi energy fixed collimator and because V200 is limited to 113 keV photon energy. Thus, it seems essential to perform clinical assessments of coming new developments, analog to Vergnaud et al. validating the use of V200 for ^177^Lu monitoring only using the 113 keV peak [23].

### Limitation

Despite being aware of the potential bias induced by statistical differences in term of accrued counts, we opted to comply with NEMA methodology that defines a scan time and specific background activity. With the ^99m^Tc-phantom, only unexpected manipulation delay prevented us to remain within the ±5% activity stipulated by the standard. However, we assume the variation of activity concentration in the background did not significantly impact our results.

Our study faced other several limitations. First, it was limited to two types of radionuclides, and we assumed the dedicated clinical protocols were optimized for this study. Then, nAC imaging was not evaluated, whereas it is widely practiced for ^99m^Tc on non-oncological indications, where use of CT is not considered clinically required due to ALARA principles. Also, some ^99m^Tc protocols also look for “cold lesions” such as in cardiac imaging, but our phantom did not contain a cold insert. Finally, specific imaging modes such as focus mode [7, 42] or enhanced reconstructions dedicated to bone scans [43, 44] were not explored.

## Conclusion

For the first time, three 360CZT-SPECT cameras have been compared through a phantom-based study under clinical conditions. From a unique acquisition, volumetric sensitivity and energy resolution were computed from raw spectra while conventional and innovative metrics ensured an objective description of reconstructed images following the vendor’s recommended approach.

We highlighted that VERITON cameras were more sensitive than StarGuide and V400 provided enhanced energy resolution. However, all three cameras had equivalent potential for ^99m^Tc imaging, while StarGuide and V400 had higher potential than V200 for ^177^Lu imaging thanks dual 113-208 keV peak imaging. We showed that dedicated visual or quantitative reconstructions offered better specific performances compared to the single visual/quantitative reconstructions inspired from PET imaging. This study did not provide an exhaustive evaluation as it was limited to two isotopes, and because nAC reconstructions, cold lesions and specific modes were not covered.

## Abbreviations

^177^Lu: 177-Lutetium
^99m^Tc: 99m-Technetium
aFOV: Axial field of view
AC: Attenuation correction
BSREM: Block-sequential regularized expectation maximization
CT: Computed tomography
CZT: Cadmium zinc telluride
DEW: Double energy window
GEHC: General electric healthcare
MTF: Modulation transfer function
MRT: Molecular radiation therapy
NM: Nuclear medicine
NPS: Noise power spectrum
NR / nNR: Noise regulation / non noise regulation
OSEM: Ordered subset expectation maximization
PET: Positron emission tomography
PSF: Point spread function
QC: QClear
RDP: Relative difference penalty
RR / nRR: Resolution recovery / non resolution recovery
SC / nSC: Scatter correction / non scatter correction
SDM: Spectrum dynamics medical
SPECT: Single photon emission tomography
TEW: Triple energy window
TTF: Task transfer function
V200: VERITON 200
V400: VERITON 400
